# Supplementary material for: Vanishing weekly hydropeaking cycles in American and Canadian rivers
Source: Nat Commun. 2021 Dec 9;12:7154. doi: 10.1038/s41467-021-27465-4 (PMC8660798; doi:10.1038/s41467-021-27465-4)
Supplement: Supplementary file 1 — Supplementary Information [file 41467_2021_27465_MOESM1_ESM.pdf]

Vanishing weekly hydropeaking cycles in American and Canadian rivers

Supplementary Information

Stephen J. Déry[1,*], Marco A. Hernández-Henríquez[1], Tricia A. Stadnyk[2], and Tara J. Troy[3]

[1]Department of Geography, Earth and Environmental Sciences, University of Northern British Columbia, Prince George, British Columbia, Canada

[2]Department of Geography, University of Calgary, Calgary, Alberta, Canada

[3]Department of Civil Engineering, University of Victoria, Victoria, British Columbia, Canada

*Corresponding author: Stephen Déry (sdery@unbc.ca)

Supplementary Table 1. Statistics on the distribution of the WHI values across 500 sites in the USA and Canada, 1980-2019. An application of the Shapiro-Wilk test to the WHI data (n = 500) suggests the distribution is not Gaussian.

Minimum	-3.168
1st Quantile	-0.398
Median	0.056
Mean	0.183
3rd Quantile	0.790
Maximum	3.783
Standard Deviation	1.121
Shapiro-Wilk test result	W = 0.973, $p = 7.19 \times 10^{-8}$

Supplementary Table 2. List of sites with the top ten ranking WHI values, 1980-2019.

Rank	Site	WHI
1	Smith R. near Philpott (VA)	3.783
2	Chattahoochee R. at Buford Dam (GA)	3.299
3	Chattahoochee R. at West Point (GA)	3.276
4	Etowah R. (GA)	3.233
5	Colorado R. at Hoover Dam (AZ/NV)	3.222
6	Smith R. at Martinsville (VA)	3.105
7	Wallenpaupack Cr. (PA)	2.934
8	Nelson R. (MB)	2.916
9	Niagara R. (NY/ON)	2.900
10	Colorado R. at Lees Ferry (AZ)	2.844

AZ: Arizona, GA: Georgia, MB: Manitoba, NV: Nevada, NY: New York, ON: Ontario, PA: Pennsylvania, VA: Virginia

Supplementary Table 3. Number and percentage of sites in 10 WHI bins in increments of 0.75 for all sites, the USA, and Canada, 1980-2019. WHI bins follow those used in Figure 1.

WHI Bin	Sites – All	Sites – All (%)	Sites – USA	Sites – USA (%)	Sites – Canada	Sites – Canada (%)
< -3.00	2	0.4	0	0.0	2	1.3
-3.00 to -2.25	9	1.8	0	0.0	9	5.9
-2.25 to -1.50	22	4.4	2	0.6	20	13.2
-1.50 to -0.75	48	9.6	23	6.6	25	16.4
-0.75 to 0.00	151	30.2	113	32.5	38	25.0
0.00 to 0.75	140	28.0	118	33.9	22	14.5
0.75 to 1.50	65	13.0	46	13.2	19	12.5
1.50 to 2.25	36	7.2	26	7.5	10	6.6
2.25 to 3.00	21	4.2	14	4.0	7	4.6
> 3.00	6	1.2	6	1.7	0	0.0

Supplementary Table 4. Alphabetical list of 14 reservoirs with gauging sites on rivers part of our database (information sourced from Ferrazzi et al.[1]).

Reservoir	River	Capacity (Mm^3)	Type*
Allegheny	Allegheny	1,460	FPAQRW
Cannonsville	WB Delaware	362	S
Carters	Coosawattee	583	FP
Cave Run	Licking	757	FQRW
Green	Green	892	FSAQR
Mark Twain	Salt	1,760	FNPRSW
Perry	Delaware	950	FSRWX
Philpott	Smith	393	FPR
Pomme de Terre	Pomme de Terre	802	FRWX
Raystown	Juniata	940	FPRW
Shelbyville	Kaskaskia	844	FSNRW
Stockton	Sac	2,060	FPRW
Waterbury	Little	46	FRP
Zoar	Housatonic	33	P

*Reservoir functions are: flood control (F), urban water supply (S), hydropower production (P), low flow augmentation (A), navigation (N), wildlife preservation (W), water conservation and sedimentation (X), water quality control (Q), and public recreation (R).

Supplementary Table 5. Ranges for the gauged area bins and 1980-2019 mean annual discharge bins illustrated in Supplementary Fig. 14 **d** and **e**.

Bin #	Gauged Area (A, km^2)	Mean Annual Discharge (Q, km^3 yr^{-1})
1	$0 < A \leq 500$	$0 < Q \leq 0.5$
2	$500 < A \leq 1,000$	$0.5 < Q \leq 1$
3	$1,000 < A \leq 5,000$	$1 < Q \leq 5$
4	$5,000 < A \leq 10,000$	$5 < Q \leq 10$
5	$10,000 < A \leq 50,000$	$10 < Q \leq 50$
6	$50,000 < A \leq 100,000$	$50 < Q \leq 100$
7	$100,000 < A \leq 500,000$	$Q > 100$
8	$500,000 < A \leq 1,000,000$	
9	$A > 1,000,000$	

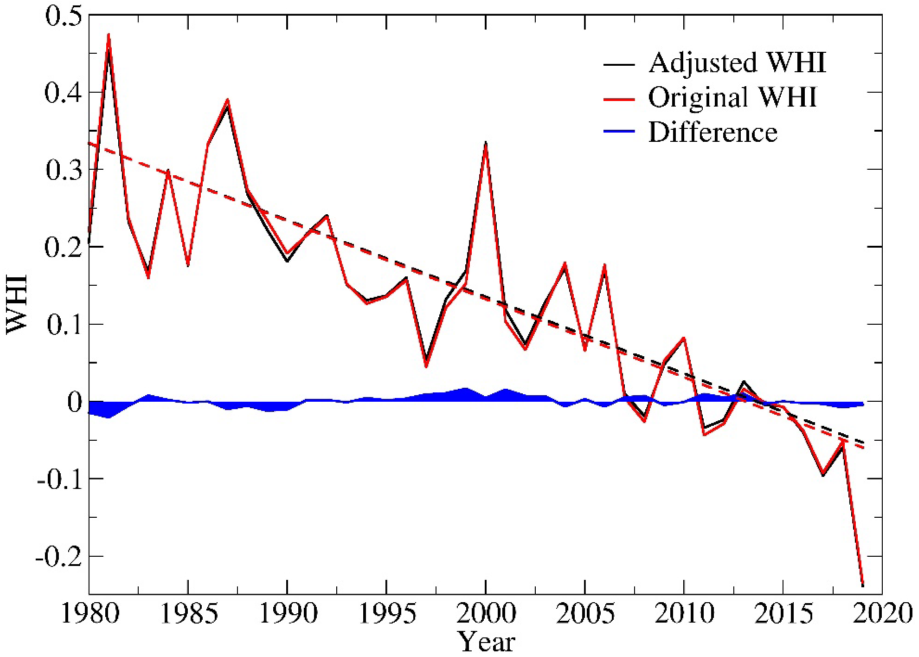

Supplementary Figure 1 The adjusted and original annual mean WHI values and their difference for 500 sites in the USA and Canada, 1980-2019. Thick dashed lines denote linear trends inferred from the Mann-Kendall test (both significant with $p < 0.05$).

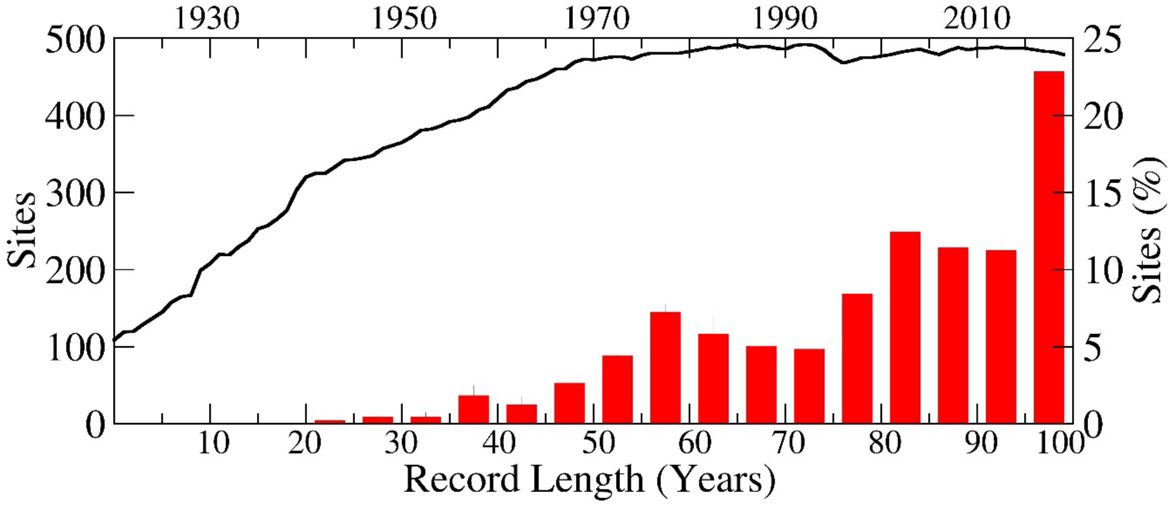

Supplementary Figure 2 Total number of sites (black line and left y-axis) used for analysis each year (top x-axis) with ≤10% missing data, 1920-2019. Red bars denote percentages of sites (right y-axis) with record lengths in bins of 5 years (bottom x-axis).

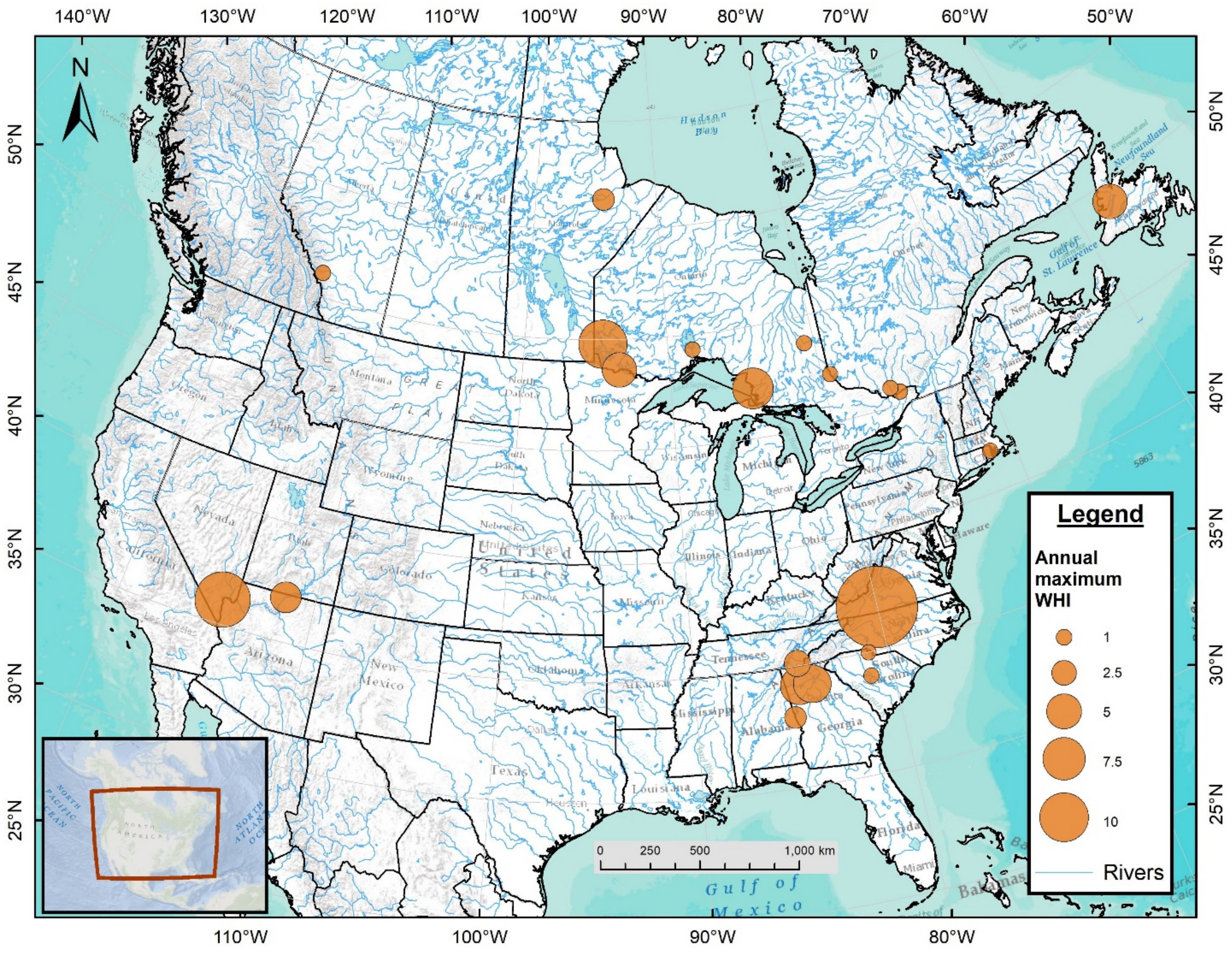

Supplementary Figure 3 Map showing the 21 sites with annual peak WHI values, 1920-2019. Circle size corresponds to the number of times a given site tops the list. Among the top ranking sites are the Smith River at Philpott (28 times), Colorado River at Hoover Dam (13 times), the Chattahoochee River at Buford Dam (12 times), the Winnipeg River at the outlet of Lake of the Woods (10 times) and the Montreal River that drains to Lake Superior (10 times).

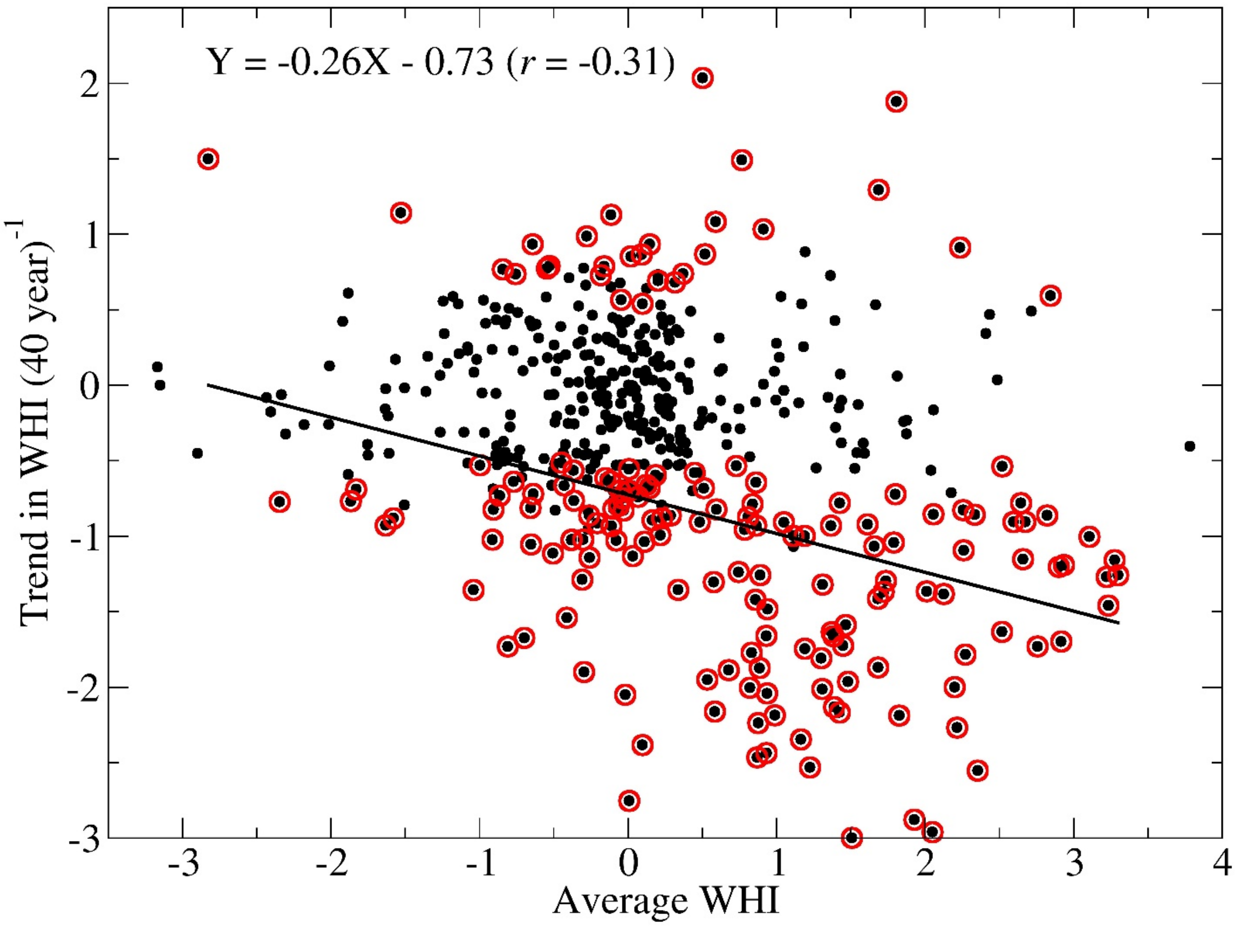

Supplementary Figure 4 Comparison of the average and trend in WHI for 479 sites with n_y ≥30 years, 1980-2019. The 479 sites are shown with black dots and the 166 red circles indicate sites with locally statistically-significant trends ($p < 0.05$). The linear regression (black line) is only based on the 166 sites with $p < 0.05$ and the Pearson correlation coefficient (r) is statistically-significant ($p < 0.05$).

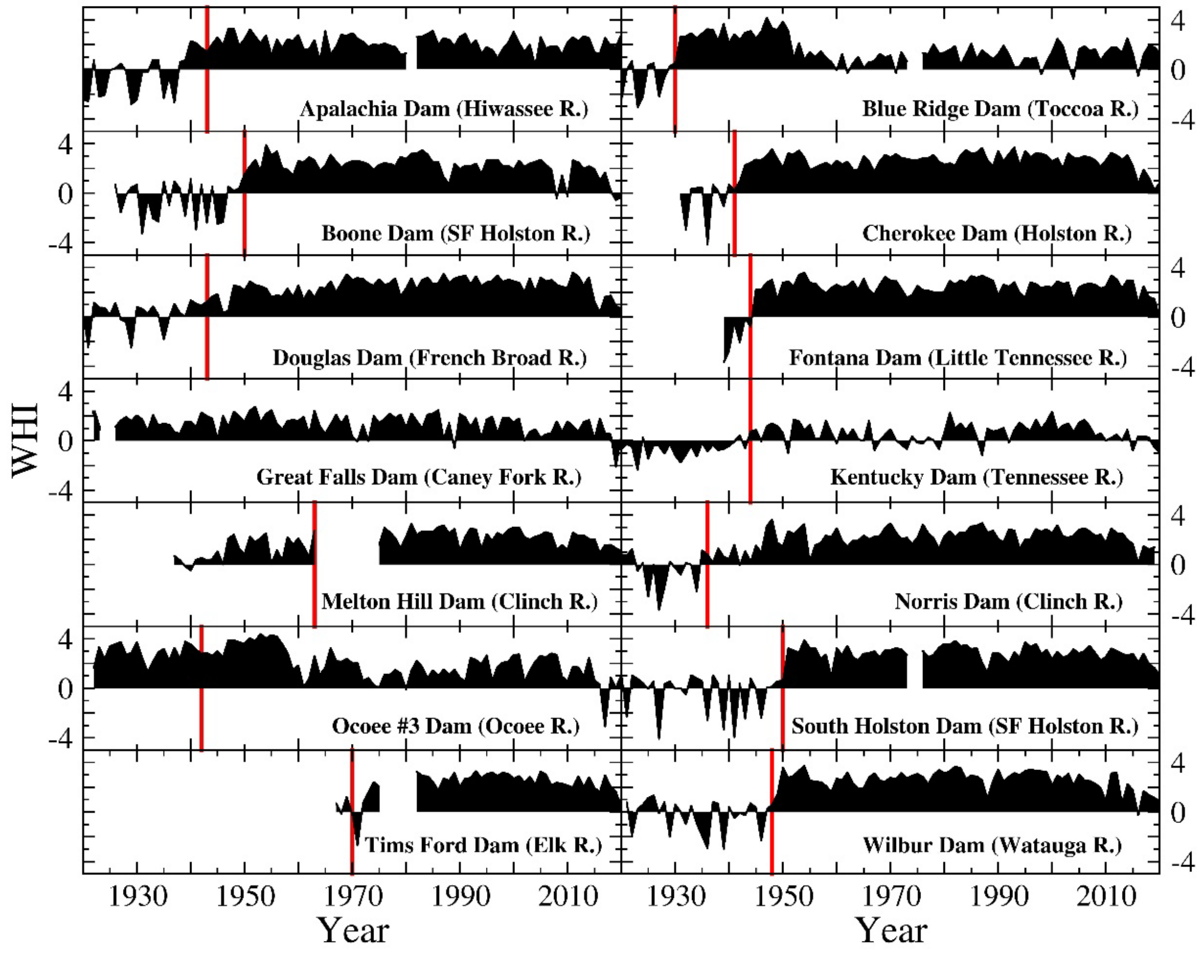

Supplementary Figure 5 Temporal evolution of annual WHI at 14 sites with hydropower dams managed by the Tennessee Valley Authority, 1920-2019. Vertical red lines denote commissioning years of hydropower dams at the gauging site (https://www.tva.com/energy/our-power-system/hydroelectric). Note that Great Falls Dam on the Caney Fork River was commissioned in 1916 explaining the absence of a vertical red line in that panel.

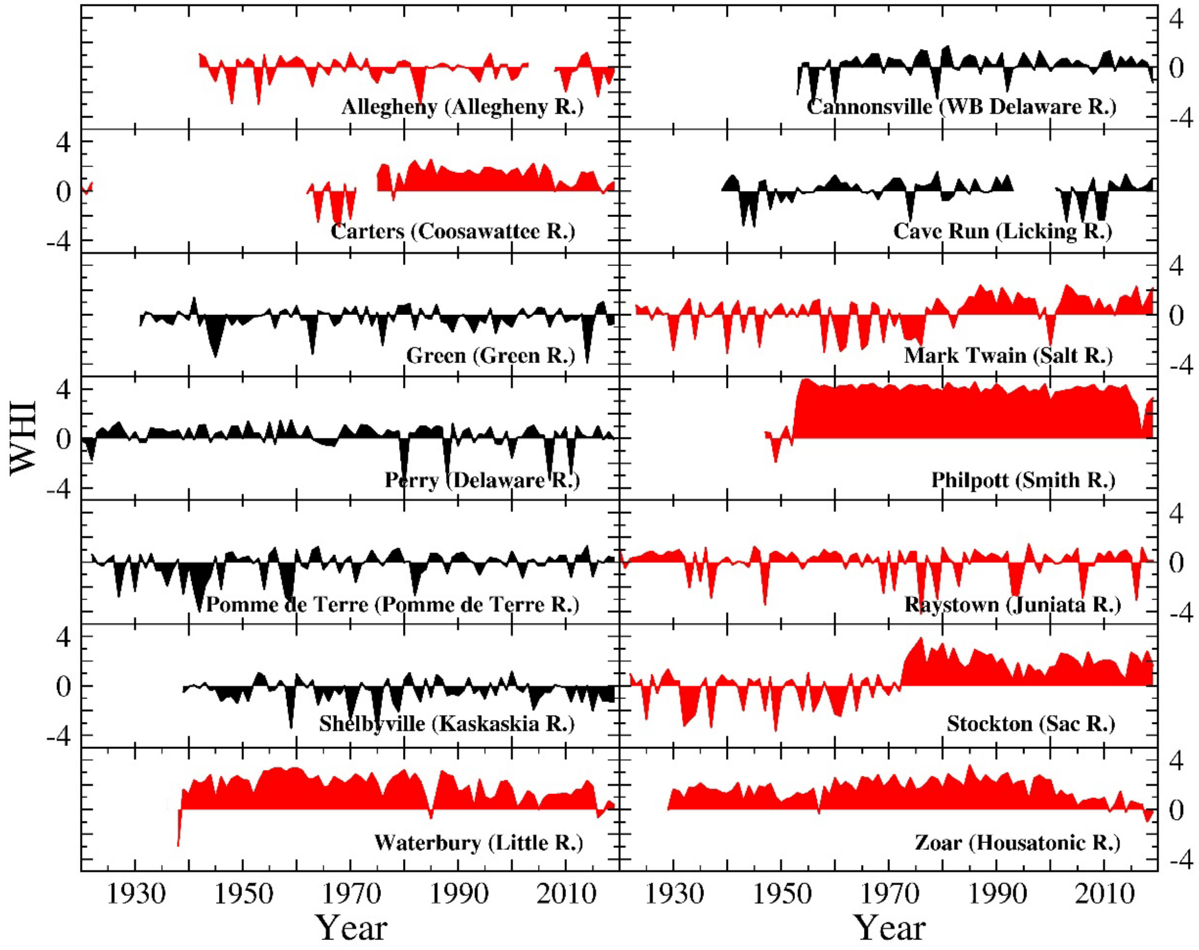

Supplementary Figure 6 Temporal evolution of annual WHI at 14 sites with upstream reservoirs with different functions (see Supplementary Table 4), 1920-2019. Red lines denote sites with an upstream reservoir managed, at least in part, for hydroelectricity production.

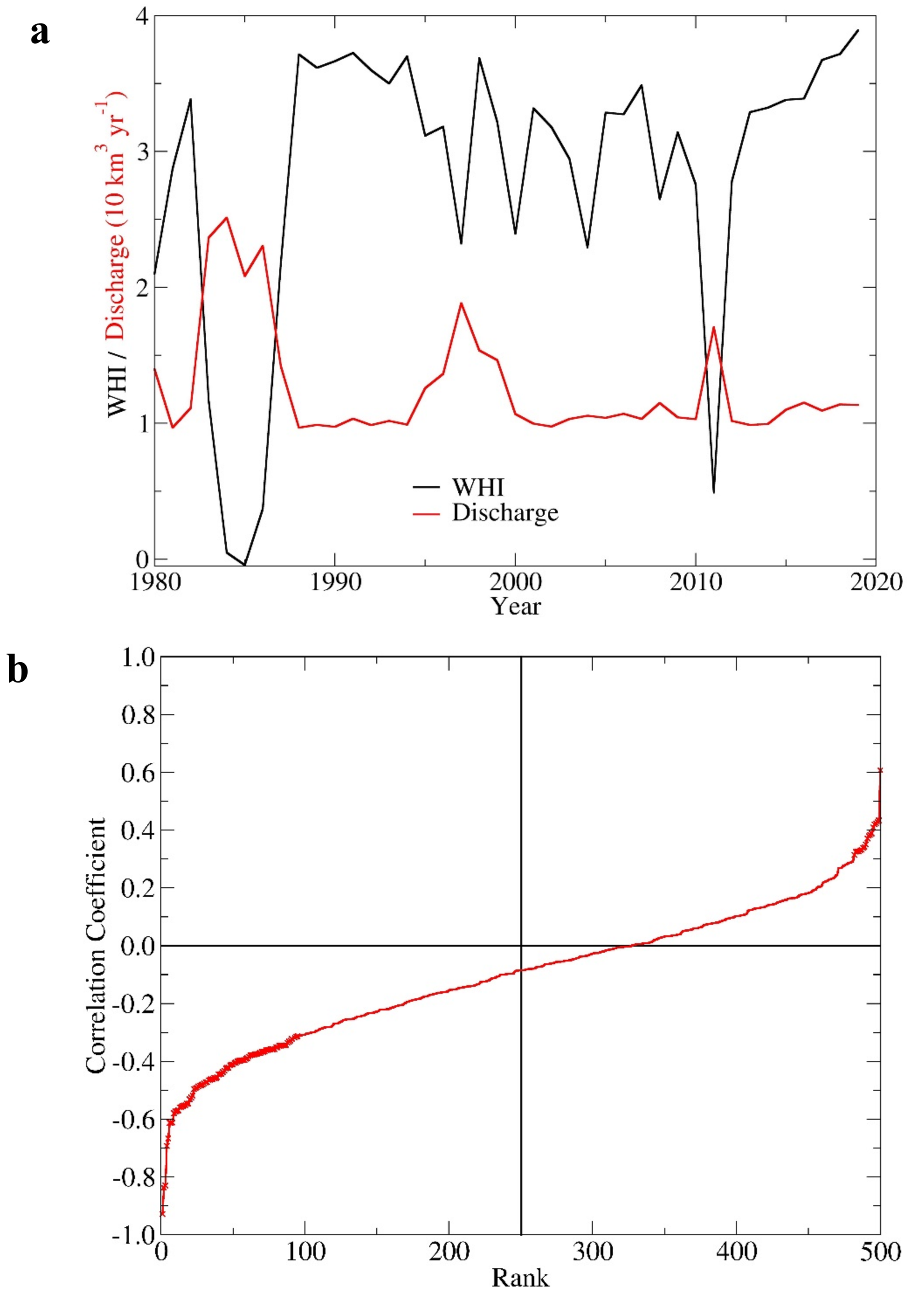

Supplementary Figure 7 a Time series of the mean annual WHI and discharge for the Colorado River at Lees Ferry, 1980-2019. **b** Pearson correlation coefficient between the 1980-2019 annual river discharge and the corresponding WHI for 500 sites across the USA and Canada. Correlation values are ranked from smallest to largest values and statistically-significant ($p < 0.05$) correlations are shown with red x symbols.

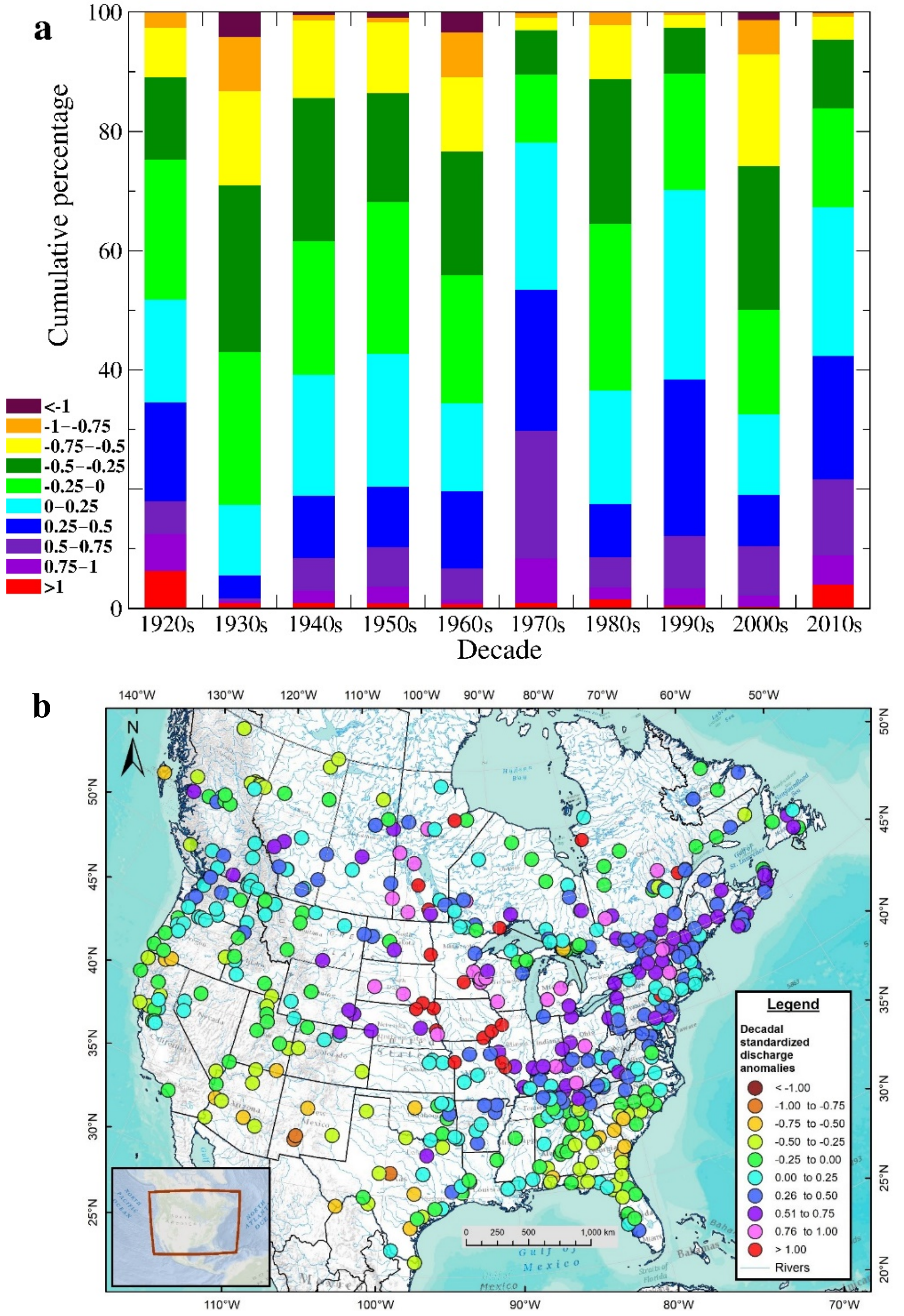

Supplementary Figure 8 a Binned distributions (1920s to 2010s) of decadal standardized anomalies in river discharge for 500 sites across the USA and Canada. Bins are in increments of 0.25 standardized discharge anomalies with the red bars indicating a standardized anomaly > 1 starting at a zero cumulative percentage and the maroon bars denoting a standardized anomaly < -1 up to 100% cumulative percentage. **b** Spatial distribution of the decadal standardized discharge anomalies at 500 sites across the USA and Canada in the 2010s using a color palette similar to panel **a**.

a

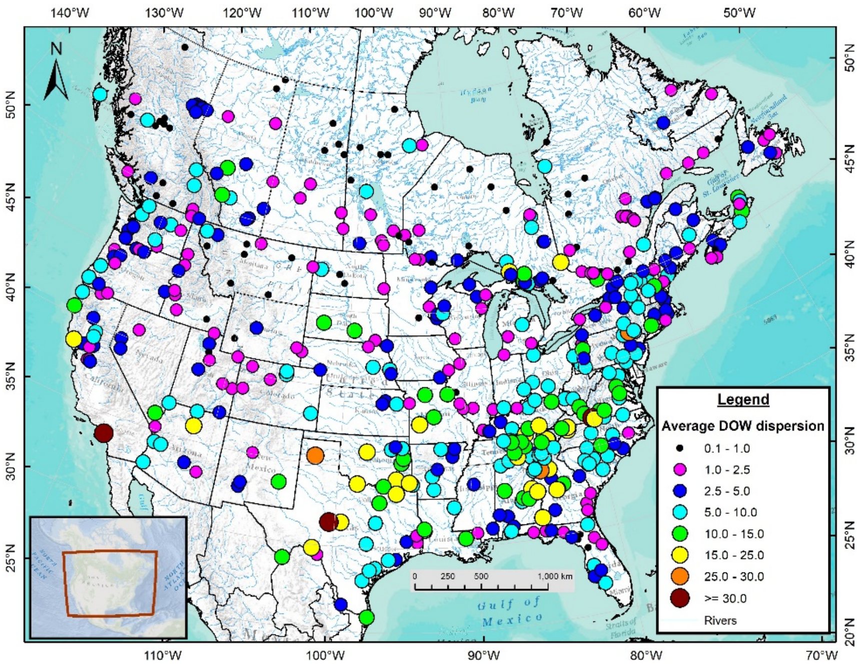

b

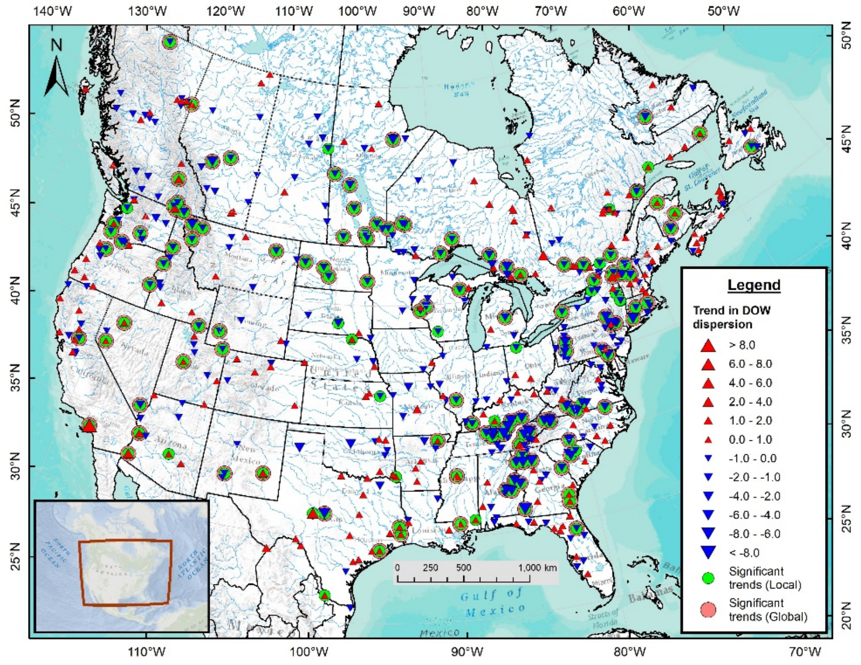

Supplementary Figure 9 a Map showing the average dispersion of day-of-the-week (DOW) flows for 500 sites across the USA and Canada, 1980-2019. Circle size corresponds to the average DOW dispersion. **b** Map illustrating monotonic trends in dispersion of DOW flows for 479 sites (with $n_y \geq 30$ years) across the USA and Canada for 1980-2019. Red upward (blue downward) pointing triangles indicate positive (negative) trends. Trend magnitudes are proportional to the triangle sizes and green circles (pink outlines) indicate locally (globally) statistically-significant trends ($p < 0.05$).

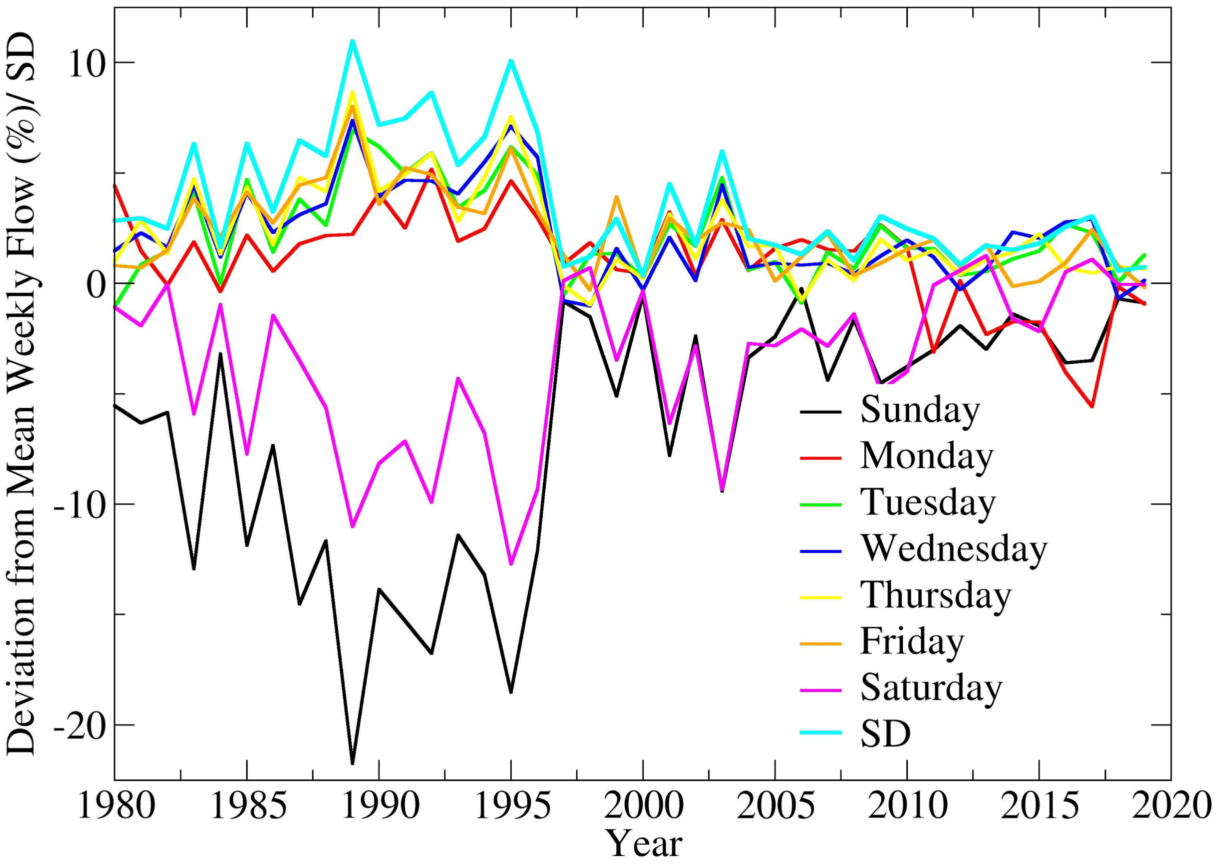

Supplementary Figure 10 Time series of deviations (%) from mean annual flows for each day of the week for the Churchill River at Churchill Falls Powerhouse, 1980-2019. Also plotted is the standard deviation (SD) in the deviations (cyan) across all seven days of the week for each year.

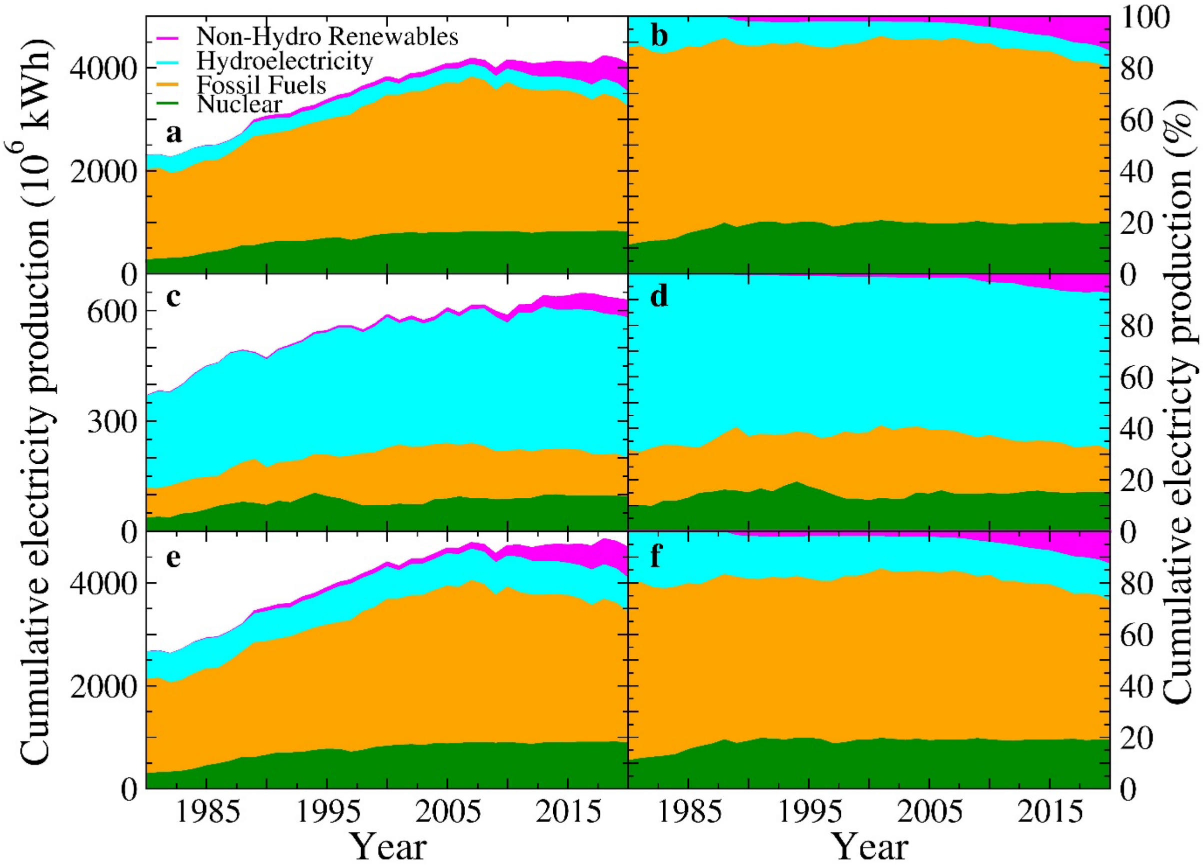

Supplementary Figure 11 Annual cumulative electricity generation (kWh, left or %, right) for four types of electricity production in **a**, **b** the USA, **c**, **d** Canada, and **e**, **f** the USA and Canada combined, 1980-2020. Note the different y-axis scales in panels **a**, **c** and **e**. There is a rapid expansion of non-hydro renewable sources of electricity in the 2010s across all regions. Data are sourced from the U.S. Energy Information Administration (http://eia.gov).

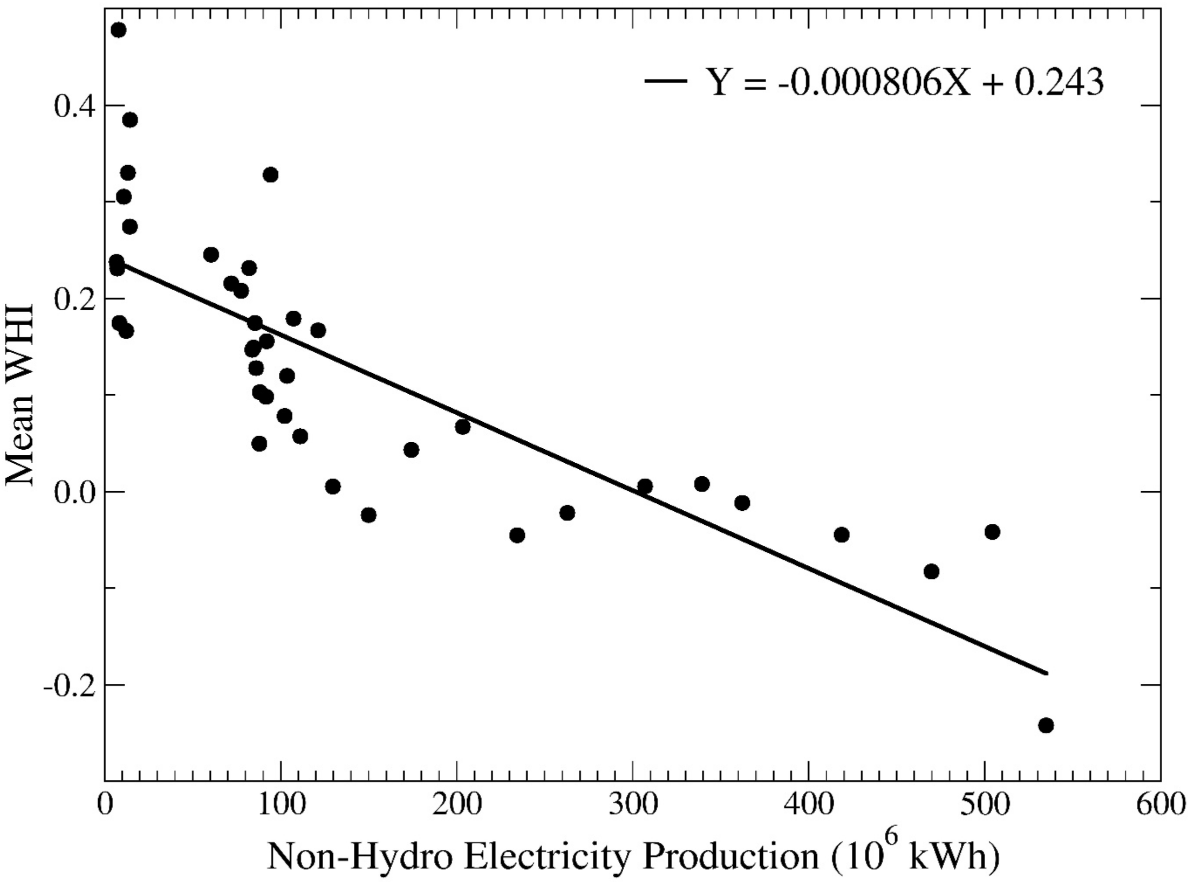

Supplementary Figure 12 Annual non-hydro electricity production across the USA and Canada combined vs. the mean annual WHI at 500 sites, 1980-2019. The thick line denotes the linear regression with r = -0.82, p < 0.05.

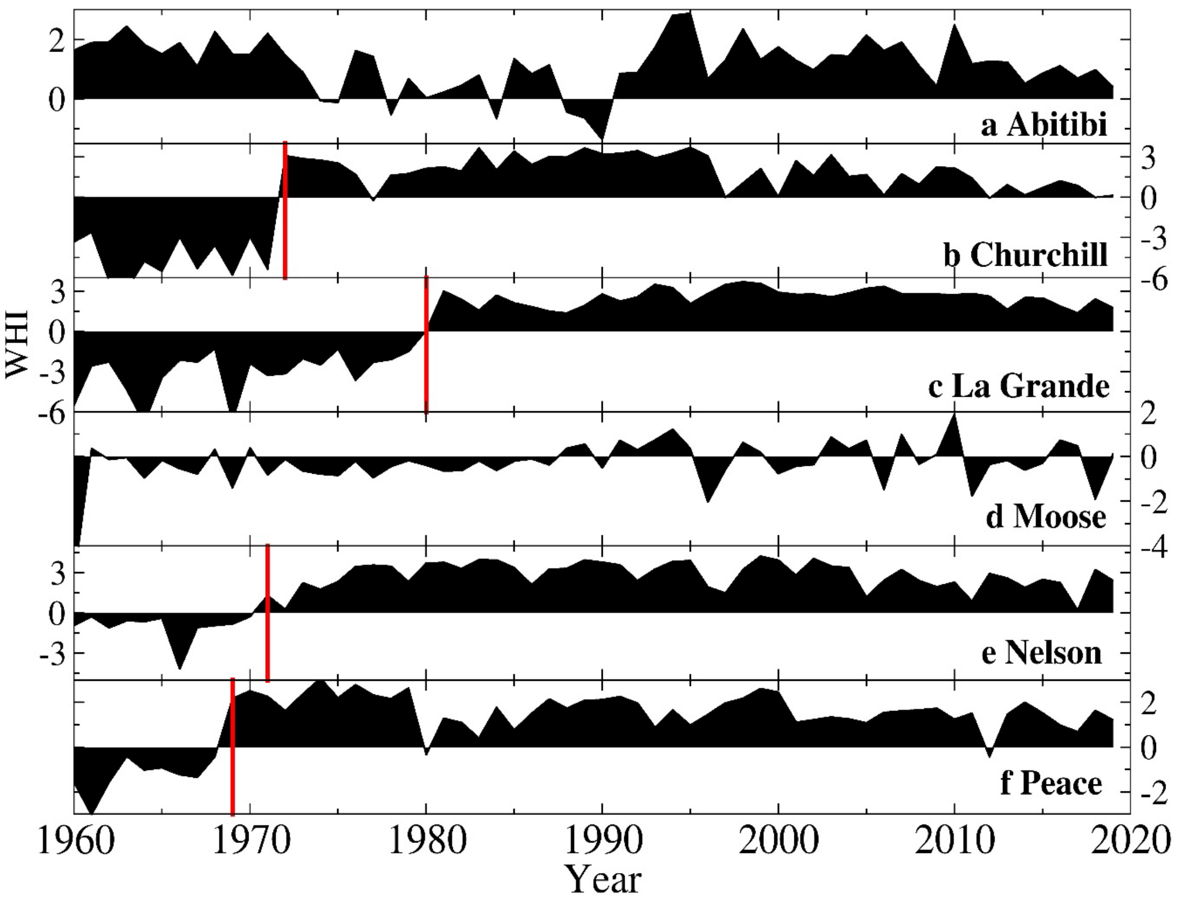

Supplementary Figure 13 Time series of the 1960-2019 WHI annual values for the **a** Abitibi River (at Onakawana), **b** Churchill River (at Churchill Falls Powerhouse, Labrador), **c** La Grande Rivière, **d** Moose River, **e** Nelson River, and **f** Peace River (at Hudson Hope). Vertical red lines denote years when hydroelectric facilities were commissioned at or just upstream of the hydrometric gauges. Sudden shifts from negative to positive WHI values follow the commissioning of hydropower generating stations at the Churchill Falls Powerhouse in 1972, the James Bay Hydroelectric Complex within the La Grande Rivière watershed in 1980, the Nelson River in 1971, and the WAC Bennett Dam in 1969. Note that y-scales vary between panels but the polygon baseline equals zero in each panel.

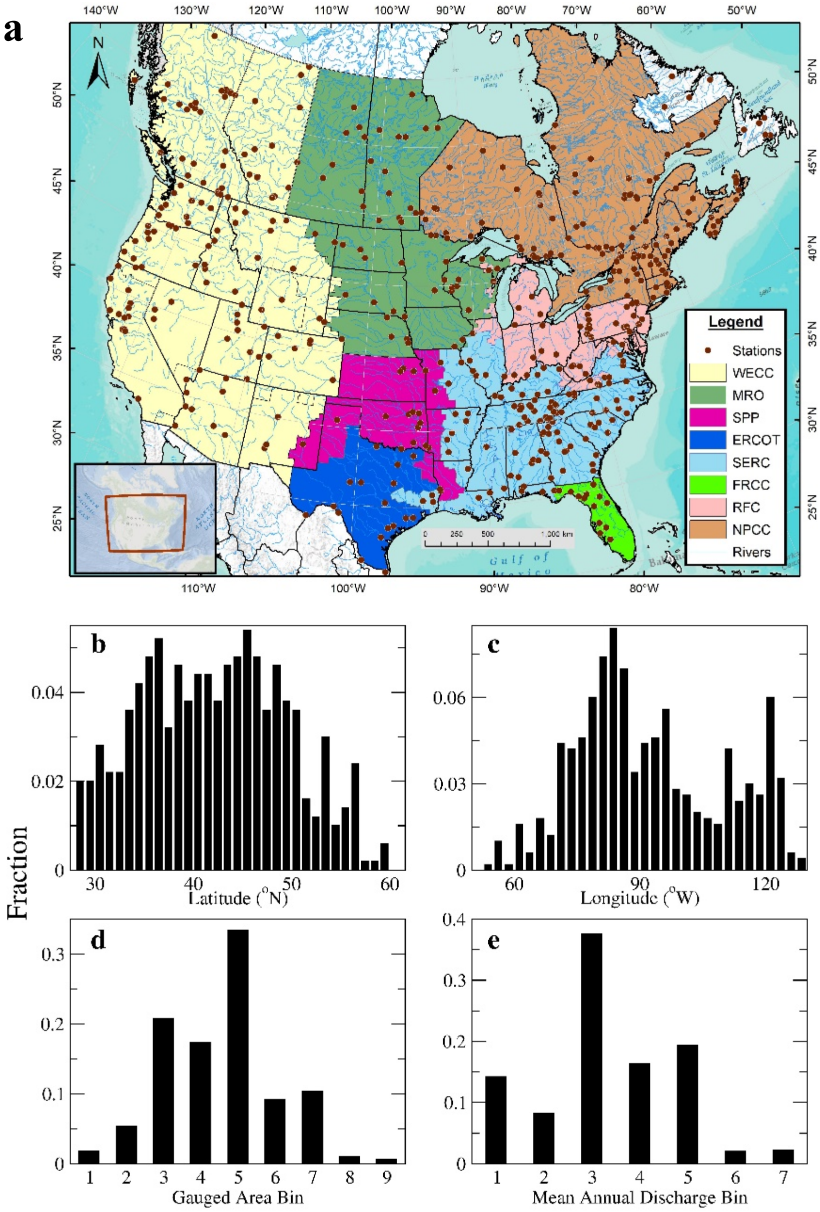

Supplementary Figure 14 a Map showing the geographical locations of the 500 sites across the USA and Canada and the primary power grid interconnections (Western Interconnection – WECC: Western Electricity Coordinating Council; Eastern Interconnection – MRO: Midwest Reliability Organization; SPP: Southwest Power Pool; SERC: SERC Reliability Corporation; FRCC: Florida Reliability Coordinating Council; RFC: Reliability First Corporation; NPCC: Northeast Power Coordinating Council; Texas Interconnection – ERCOT: Electrical Reliability Council of Texas). The fraction of the 500 sites distributed by **b** latitude (°N), **c** longitude (°W), **d** gauged area bin, and **e** 1980-2019 mean annual discharge bin. See Supplementary Table 5 for the ranges for the gauged area bins and 1980-2019 mean annual discharge bins.

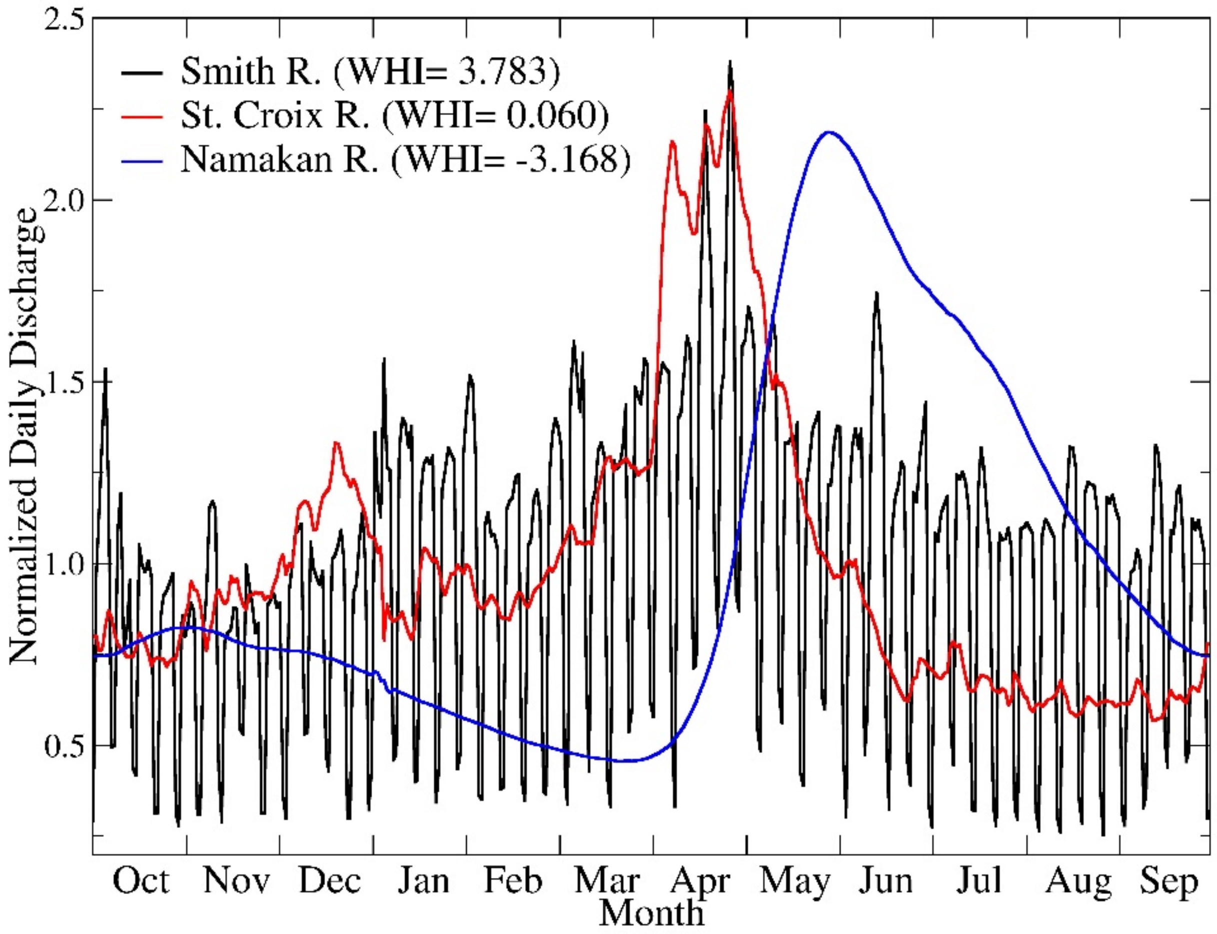

Supplementary Figure 15 The 1980-2019 normalized, average daily discharge considering the day of the week for the Namakan River (Minnesota/Ontario), St. Croix (Maine/New Brunswick), and Smith River near Philpott (Virginia) illustrates the WHI ranging from the minimum (-3.168), median (0.060), and maximum (3.783) values, respectively.

Supplementary Methods

Assessment of uncertainty. Uncertainty in the results arises from at least four sources: 1) potential errors in the discharge measurements; 2) in-filling of temporal gaps; 3) data homogeneity; and 4) serial correlation on trend analyses. These issues are addressed using the following approaches.

1. Discharge measurement errors. Measurements of river discharge may accrue errors due to various issues including from malfunctioning equipment, human, transcription and random errors, and inaccuracies of rating curves. In turn, this leads to potential errors of ±2-5% in the discharge data[2]. To assess the potential role of such errors on the weekly hydropeaking index (WHI) results, we conduct here eight additional experiments to test the sensitivity of our results to perturbed discharge data. The WHI is computed over 1980-2019 at all 500 sites with a random error added or subtracted to each value of daily river discharge used for analysis. The random error ranges either from ±2% or ±5% and follows either a uniform or a Gaussian distribution. Based on these eight additional experiments, the overall minimum and maximum WHI for each of the 500 sites is retained for comparison with the original value (Supplementary Figure 16).

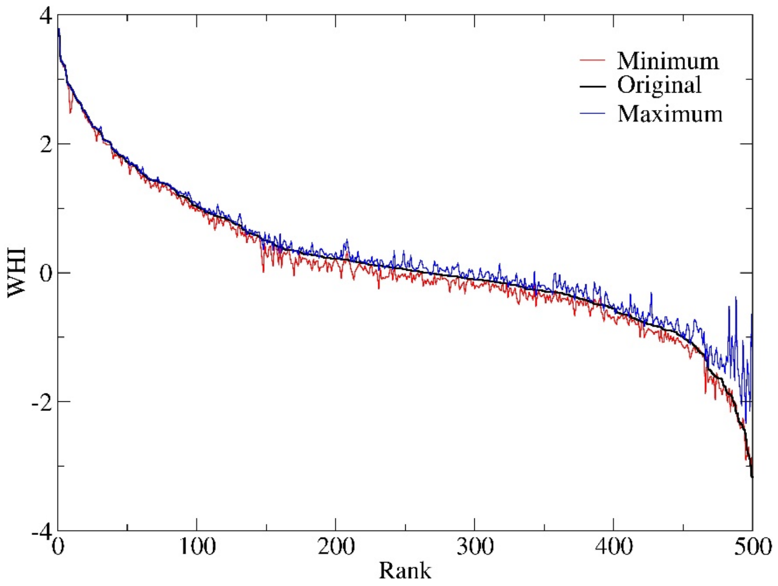

Supplementary Figure 16 The 1980-2019 minimum and maximum WHI at 500 sites based on eight sensitivity experiments in which random errors are added/subtracted to the original daily discharge data. Results are compared to the 1980-2019 original WHI value ranked in descending order.

These results show that there is little discrepancy between the original WHI values and the range obtained from the sensitivity experiments aside from those with large negative values (WHI < -2). This suggests the WHI results, particularly for the hydropeaking sites, are quite robust and relatively insensitive to random errors in discharge measurements; however, care must be taken in interpreting sites with large negative WHI given their greater sensitivity to the input data.

2. In-filling strategy. Aside from measurement errors, uncertainty in the results may arise from the gap-filling strategy. In this study, we make use of the available discharge data in any given calendar year so long as < 10% of the data are missing. In years with < 10% missing data, gaps are in-filled with the mean daily discharge over the period of record at that site. Supplementary Data 2 lists the percentage of missing data in-filled at

all 500 sites, with the maximum being 0.58%. Thus in two additional sensitivity experiments, we substitute 0.58% (one measurement at 172 day intervals) and 0.29% (one measurement at 345 day intervals) of the original daily discharge data at all 500 sites with the mean daily discharge of each site's respective period of record.

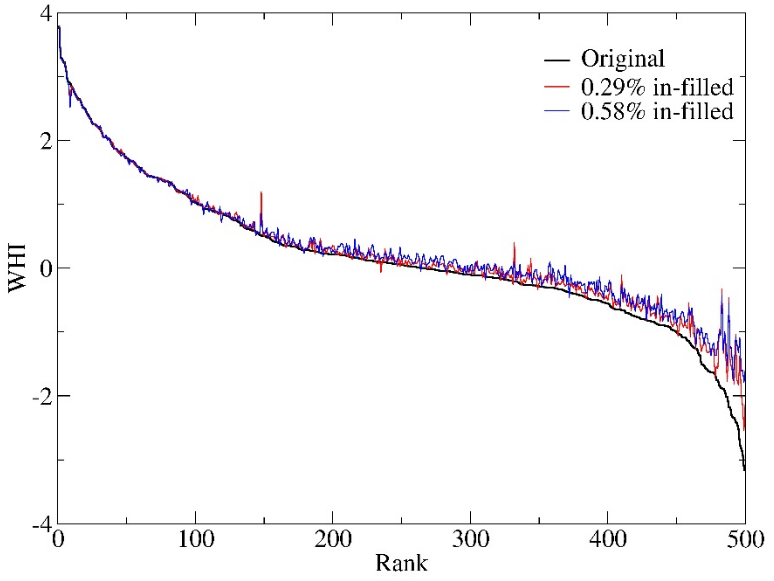

Supplementary Figure 17 The 1980-2019 minimum and maximum WHI at 500 sites based on two sensitivity experiments in which 0.58% and 0.29% of the original daily discharge data are in-filled with the period of record mean daily discharge. Results are compared to the 1980-2019 original WHI value ranked in descending order.

Supplementary Figure 17 illustrates the increasing influence of missing data on the results as the WHI decreases. Similar to the potential effects of measurement errors, the largest discrepancies between the original WHI and values from the two additional sensitivity experiments arise when WHI < -2. Overall, gap-filled data tend to increase the WHI values but with minimal differences between the gap-filled time series when WHI > 0.

3. Data homogeneity. At a few sites (e.g. the Nelson, Richelieu and Saguenay rivers), the river discharge time series are developed by splicing records from different hydrometric gauges. In some cases, this is due to a slight relocation of a hydrometric gauge or the inception of new points of regulation along a waterway. This approach is preferred in generating longer records representative of these waterways over the possible inhomogeneity introduced in the records and associated uncertainty in the WHI results. To verify the sensitivity of the results to this approach we computed the WHI for the Nelson River during 1990-2019 for which we have overlapping daily discharge data at three proximal sites, moving downstream: the Kettle, Long Spruce and Limestone generating stations (Supplementary Figure 18).

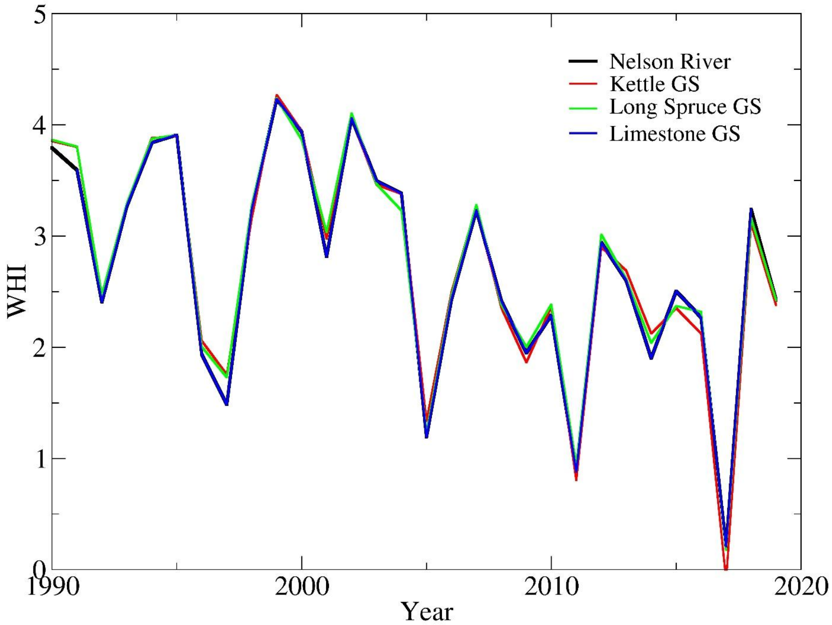

Supplementary Figure 18 Comparison of the original WHI time series for the Nelson River with that computed for the Kettle, Long Spruce and Limestone generating stations (GS), 1990-2019.

Using the Nelson River as an example, we observe minimal difference in the annual WHI values between three adjacent hydroelectric facilities. The hydropower

infrastructures on the lower Nelson River (downstream of Stephens Lake) are run-of-river and operate in a similar fashion. As the Limestone generating station came online only in 1990, we use discharge data from the upstream Long Spruce generating station site first prior to 1990, and when unavailable, the data from the upstream Kettle generating station. Irrespective of the site used, however, the annual WHI values diverge marginally. Prior to the commissioning of the Kettle generating station in 1971, we use discharge data at the Kelsey generating station upstream of Stephens Lake. At the Kelsey gauge, influence from upstream reservoir operations is quite different from that at the three generating stations downstream of Stephens Lake, leading to much lower WHI values (< 0) on other sections of the Nelson River (Supplementary Figure 13). In any case, data inhomogeneity owing to splicing data records is not likely to be a significant issue in the results for which multiple hydrometric gauges or sites are used to construct the daily discharge time series.

4. Serial correlation on trend analyses. Serial correlation in the WHI time series may lead to overestimation of the trend magnitudes and their significance. While there remains some debate as to whether hydrological time series should be pre-whitened or not prior to trend analysis[3], we assess here whether autocorrelation is a major influence on our trend results. We follow Yue et al.[4] in pre-whitening the 1980-2019 WHI time series when the Mann-Kendall test (MKT) yields a statistically-significant ($p < 0.05$) trend on the original data. We detect a statistically-significant lag-1 autocorrelation in detrended WHI time series at 26 sites where the MKT trends are also significant. After pre-whitening the WHI time series, we find that only one trend value is no longer

significant: the English River at Manitou Falls in Ontario (negative trend). This suggests that lag-1 autocorrelation effects on the ability to detect trends in the WHI time series remain minimal, with 165 sites retaining statistically-significant WHI trends when pre-whitening is considered.

Several studies[5-8] suggest that long-term persistence (beyond lag-1 autocorrelation) may also lead to overestimation of trend significance in hydrometeorological variables. Further analysis reveals that only six sites with statistically-significant trends also exhibit autocorrelations with lag-2 or higher with $p < 0.05$ in their detrended WHI time series. Thus care is required when interpreting the significance of the trends for the Cowlitz, Michipicoten, Montreal (Lake Superior), Obey, Sacandaga and Tallapoosa rivers given the presence of long-term persistence in their WHI time series.

Conclusion. This study uncovers a general pattern towards vanishing weekly hydropeaking cycles across the USA and Canada. These findings rely on river discharge measurements at 500 sites collected by a variety of sources. While the hydrological data are generally subject to rigorous quality analysis and control by the collecting agency, they remain subject to errors that can lead to uncertainty in the results reported in this study. Our assessment of the influence of potential random errors in the daily discharge measurements, of the gap-filling procedure, data inhomogeneity and autocorrelation on the trend analyses suggests our primary results remain robust. Random errors and temporal gap filling yield greater uncertainty at very low WHI values (< -2); however, given this study focuses on weekly hydropeaking, this is less of a concern and does not influence our main conclusion. Imposing random

errors or in-filling data for sites with robust annual cycles (e.g. the Namakan River, see Supplementary Figure 15) creates departures from the smooth hydrographs, cascading energy towards shorter (sub-annual) time scales. This is reflected by the increasing response of the frequency domain term in the WHI and thus generally augmenting the WHI for those sites. Data homogeneity and serial correlation impacts on trend analyses are also shown to have minimal influence on the results. As such we conclude that our study's main finding of vanishing weekly hydropeaking signals across the USA and Canada is robust and not an artifact of uncertain data.

Supplementary References

1. Ferrazzi, M., Woods, R. A. & Botter, G. Climatic signatures in regulated flow regimes across the Central and Eastern United States. *J. Hydrol. Reg. Studies*, **35**, 100809 (2021).

2. Lammers, R. B., Shiklomanov, A. I., Vörösmarty, C. J., Fekete, B. M. & Peterson, B. J. Assessment of contemporary Arctic river runoff based on observational discharge records. *J. Geophys. Res.* **106**, 3321-3334 (2001).

3. Bayazit, M. & Önöz, B. To prewhiten or not to prewhiten in trend analysis? *Hydrol. Sci. J.* **52**, 611-624 (2007).

4. Yue, S., Pilon, P., Phinney, B. & Cavadias, G. The influence of autocorrelation on the ability to detect trend in hydrological series. *Hydrol. Process.* **16**, 1807-1829 (2002).

5. Dinpashoh, Y., Mirabbasi, R., Jhajharia, D., Abianeh, H. Z. & Mostafaeipour, A. Effect of short-term and long-term persistence on identification of temporal trends. *J. Hydrol. Eng.* **19**, 617-625 (2014).

6. Khaliq, M. N., Ouarda, T. B. M. J. & Gachon, P. Identification of temporal trends in annual and seasonal low flows occurring in Canadian rivers: the effect of short- and long-term persistence. *J. Hydrol.* **369**, 183-197 (2009).

7. Kumar, S., Merwade, V., Kam, J. & Thurner, K. Streamflow trends in Indiana: Effects of long term persistence, precipitation and subsurface drains. *J. Hydrol.* **374**, 171-183 (2009).

8. Zamani, R., Mirabbasi, R., Abdollahi, S. & Jhajharia, D. Streamflow trend analysis by considering autocorrelation structure, long-term persistence, and Hurst coefficient in a semi-arid region of Iran. *Theor. Appl. Climatol.* **129**, 33-45 (2017).
