# Supplementary material for: Vanishing weekly hydropeaking cycles in American and Canadian rivers
Source: Nat Commun. 2021 Dec 9;12:7154. doi: 10.1038/s41467-021-27465-4 (PMC8660798; doi:10.1038/s41467-021-27465-4)
Supplement: Supplementary file 3 — Description of Additional Supplementary Files [file 41467_2021_27465_MOESM3_ESM.pdf]

Description of Additional Supplementary Files

File name: Supplementary Data 1

Description: Alphabetical list of the 500 sites across the USA and Canada with results of the Pettitt test applied to the 1980-2019 weekly hydropeaking index (WHI) time series. Listed are the test statistic U*, the corresponding *p*-value, year for the break point (BPY), and the mean WHI for the period prior to and after the break point. Pettitt test results are not available (NA) for 21 sites with $n_y < 30$ years over 1980-2019.

File name: Supplementary Data 2

Description: Alphabetical list of 500 sites across the USA and Canada including the site names, 2-character codes, gauge identification numbers, geographical coordinates, gauged area, period of data availability, percentage data in-filled, and the mean annual 1980-2019 weekly hydropeaking index (WHI).

File name: Supplementary Data 3

Description: Details of 69 sites (listed alphabetically by river name) in the USA and Canada where proprietary or unpublished data are used. Start and end years are those only for which data are acquired, although full available records are generally longer (see Supplementary Data 2). Data providers are as follows: BC Hydro, Evolugen, H2O Power, Hydro-Québec (HQ), International Boundary and Water Commission (IBWC), Manitoba Hydro (MH), Nalcor Energy, NB Power, Ontario Power Generation (OPG), Rio Tinto, Tennessee Valley Authority (TVA), TransAlta, and United States Army Corps of Engineers (USACE). CF(L)Co: Churchill Falls (Labrador) Corporation, GS: Generation Station, LG: La Grande, NL: Newfoundland and Labrador.

File name: Supplementary Data 4

Description: Time series of weekly hydropeaking index (WHI) for 500 sites in the USA and Canada, 1920-2019. Sites are listed alphabetically as in Supplementary Data 2. WHI values are not available (NA) when > 10% of the daily discharge data are missing in a given calendar year.
